# The conserved DNMT1-dependent methylation regions in human cells are vulnerable to neurotoxicant rotenone exposure

**DOI:** 10.1186/s13072-020-00338-8

**Published:** 2020-03-16

**Authors:** Dana M. Freeman, Dan Lou, Yanqiang Li, Suzanne N. Martos, Zhibin Wang

**Affiliations:** 1 Laboratory of Environmental Epigenomes, Department of Environmental Health & Engineering, Bloomberg School of Public Health, Johns Hopkins University, Baltimore, MD USA; 2 The State Key Laboratory of Biocatalysis and Enzyme Engineering, School of Life Sciences, Hubei University, Wuhan, 430062 Hubei China; 3 Department of Oncology, School of Medicine, Johns Hopkins University, Baltimore, MD USA

**Keywords:** DNA methylation, Allele-specific methylation, Rotenone, Parkinson’s disease, Neurotoxicity

## Abstract

**Background:**

Allele-specific DNA methylation (ASM) describes genomic loci that maintain CpG methylation at only one inherited allele rather than having coordinated methylation across both alleles. The most prominent of these regions are germline ASMs (gASMs) that control the expression of imprinted genes in a parent of origin-dependent manner and are associated with disease. However, our recent report reveals numerous ASMs at non-imprinted genes. These non-germline ASMs are dependent on DNA methyltransferase 1 (DNMT1) and strikingly show the feature of random, switchable monoallelic methylation patterns in the mouse genome. The significance of these ASMs to human health has not been explored. Due to their shared allelicity with gASMs, herein, we propose that non-traditional ASMs are sensitive to exposures in association with human disease.

**Results:**

We first explore their conservancy in the human genome. Our data show that our putative non-germline ASMs were in conserved regions of the human genome and located adjacent to genes vital for neuronal development and maturation. We next tested the hypothesized vulnerability of these regions by exposing human embryonic kidney cell HEK293 with the neurotoxicant rotenone for 24 h. Indeed,14 genes adjacent to our identified regions were differentially expressed from RNA-sequencing. We analyzed the base-resolution methylation patterns of the predicted non-germline ASMs at two neurological genes, *HCN2* and *NEFM*, with potential to increase the risk of neurodegeneration. Both regions were significantly hypomethylated in response to rotenone.

**Conclusions:**

Our data indicate that non-germline ASMs seem conserved between mouse and human genomes, overlap important regulatory factor binding motifs, and regulate the expression of genes vital to neuronal function. These results support the notion that ASMs are sensitive to environmental factors such as rotenone and may alter the risk of neurological disease later in life by disrupting neuronal development.

## Background

DNA methylation refers to the addition of a methyl group (CH3) to the cytosine base of DNA by DNA methyltransferases. This predominately occurs at cytosine–guanine adjacent sites known as CpG sites. For most genomic loci, DNA methylation is coordinated across both inherited alleles. However, some loci maintain CpG methylation at only one allele and these regions are described to have allele-specific methylation (ASM; previously known as differentially methylated region DMR) [6]. The most well-known of these regions are germline ASMs which control the expression of imprinted genes in a parent of origin-dependent manner. Imprinted genes are crucial in development and are commonly associated with genetic disorders such as Beckwith–Wiedemann, Angelman, and Prader-Willi syndromes [10]. In addition to the control of imprinted gene expression, DNA methylation is a key to maintain genome stability via silencing retrotransposons [11, 74].

Investigations demonstrate that two types of genomic regions, imprinted germline ASMs and intracisternal A-particle (IAP)-like retrotransposons, seem vulnerable to environmental factors. Therefore, these two regions are proposed to be pivotal for understanding human disease in response to exposure and popularly pursued in animal and epidemiological studies [32, 51]. The former is attractive because exposure altered ASMs are anticipated to be faithfully transmitted to somatic cells during rounds of global demethylation and remethylation in early embryos [5, 33]. As a result, parental exposure can be epigenetically inherited to modify offspring phenotype [22]. The latter is exemplified in mice by the bisphenol A-hypomethylated IAP at the *agouti* gene for variations of coat color and obesity, as well as by altered methylation of IAPs at *Axin*^*Fu*^ for tail kinkiness [17, 61, 83].

In our recent work, we developed two approaches, no-rescued DMRs (NORED) and methylation mosaicity analyses (MethylMosaic), to identify numerous genomic loci bearing potential ASMs [48]. Both the known imprinted germline ASMs and newly identified ASMs are dependent on DNA methyltransferase 1 (DNMT1) [44]. Many of these novel ASMs are presumably sequence (single nucleotide polymorphism; SNP)-influenced ASMs [35]. For example, in a reciprocal cross between 129S1/SvlmJ and Cast/EiJ or between C57BL/6NJ and Cast/EiJ, the Cast allele with SNP C of *Hcn2* ASM is always hypomethylated (i.e., independent of parental origin), whereas the 129 allele or the C57 allele with SNP A is always hypermethylated. Standing out of the previously appreciated sequence-dependent ASMs, a new paradigm of switchable ASMs that shows equal chances of either paternal or maternal allele to be methylated was revealed by our report [48]. Importantly, the switchable feature seems also conserved in the human genome. At the *DLGAP2* locus, independent evidence confirms a maternally imprinted ASM during pre-implantation switched to a random ASM in somatic tissues during gestation [50]. Collectively, the mouse genome or human genome contains more ASMs (including both sequence-dependent and switchable ASMs) than previously appreciated [15, 48, 50, 55]. The newly revealed random, switchable ASMs remind us of features in X chromosome inactivation, leading to a proposed hypothesis of regional autosomal chromosome inactivation [76].

Currently, germline ASMs are being increasingly considered in human disease; however, less studied are non-germline ASMs, which maintain CpG methylation at one allele independent of the parent of origin [15, 48, 82]. These regions regulate the expression of non-imprinted genes and these genes are hypothesized to have random monoallelic expression. Due to their predicted monoallelicity (DNA methylation and transcripts), we hypothesize that these regions are also targets for environmental factors and associated with disease like germline ASMs [34, 70].

The goal of this study was to determine whether our identified candidate ASMs in the mouse genome were in conserved regions of the human genome and to explore the possible adverse effects of differential methylation in these regions by examining their tissue expressivity and functional enrichment. Last, we tested our hypothesis that genes adjacent to non-germline ASMs would be vulnerable to environmental factors by exposing human embryonic kidney *HEK293* cells to the pesticide rotenone for 24 h. We used whole transcriptome RNA-sequencing and targeted bisulfite-amplicon sequencing to evaluate changes in expression of adjacent genes and DNA methylation at candidate ASMs in response to rotenone. Indeed, our data demonstrate the vulnerability of these new non-traditional ASMs to environmental exposure [23].

## Results

### DNMT1-dependent regions in the mouse and human genome

**Fig. 1:**
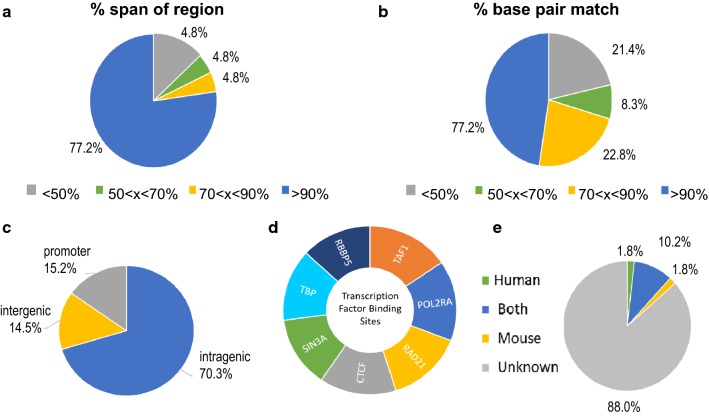
Characterization of conserved DNMT1-dependent regions in the Human genome. **a** The percent span of the DNMT1-dependent regions in mouse covered by the identified human conserved regions. Pie chart represents the percentage of all identified conserved regions in the human genome that fall into each category. **b** The percent base-pair match of the DNMT1-dependent regions in mouse with the identified human conserved regions. Pie chart represents the percentage of all conserved regions in the human genome that fall into each category. **c** The percentage of all conserved regions in the human genome that are located within the promoter, the gene body, or in non-coding intergenic regions. **d** The top transcription factor binding sites found within all human conserved regions. **e** The percentage of known germline ASMs in our conserved DNMT1-dependent regions separated by genome

Two approaches, NORED and MethylMosaic, independently identified over 2000 regions with DNMT1 dependency and allele-specific methylation. To simplify future interpretation, we focused on 207 overlapped regions (i.e., ‘NORED + MethylMosaic’ regions) to initiate our investigation. We compared these 207 regions from mouse and observed 145 of these regions were conserved in the human genome. Most regions identified in the human genome were highly conserved with > 70% matched bases for more than 90% of the entire span of the region (Fig. 1a, b). Analyzing these regions in the genome browser, we noted that 70% of the conserved regions in the human genome were located in the gene body and approximately 50% of them had transcription factor binding sites in human embryonic stem cells (Fig. 1c, d). The two transcription factors that were the most significantly enriched were POL2RA and TAF1 with binding sites at 19% of conserved DNMT1 regions. Both were concentrated around transcription start sites and regulate RNA polymerase II binding and processivity in gene transcription. The third most enriched transcription factor was CTCF with binding motifs in 17% of conserved regions and most often found within intragenic regions. The top transcription factors with binding motifs found in intergenic sites were CTCF, SIN3A, and RAD21. All three transcription factors are crucial in regulating chromatin structure to repress gene transcription and inhibition of these factors is closely associated with human disease [14, 78, 85]. We searched conserved human genes for known imprinted genes at germline ASMs using the GeneImprint database and found 20 known imprinted genes (Fig. 1e). Most of the human genes found at conserved DNMT1-dependent regions had an unknown imprinted status and thus were considered candidate non-germline ASMs. Prior examination of DNA methylation in four independent mouse embryonic stem cell lines validated our hypothesis at one conserved gene (*HCN2*) that non-germline ASMs can exhibit a random, switchable pattern [48].

### Human DNMT1-dependent genes are enriched in cellular processes associated with cell–cell signaling

**Fig. 2:**
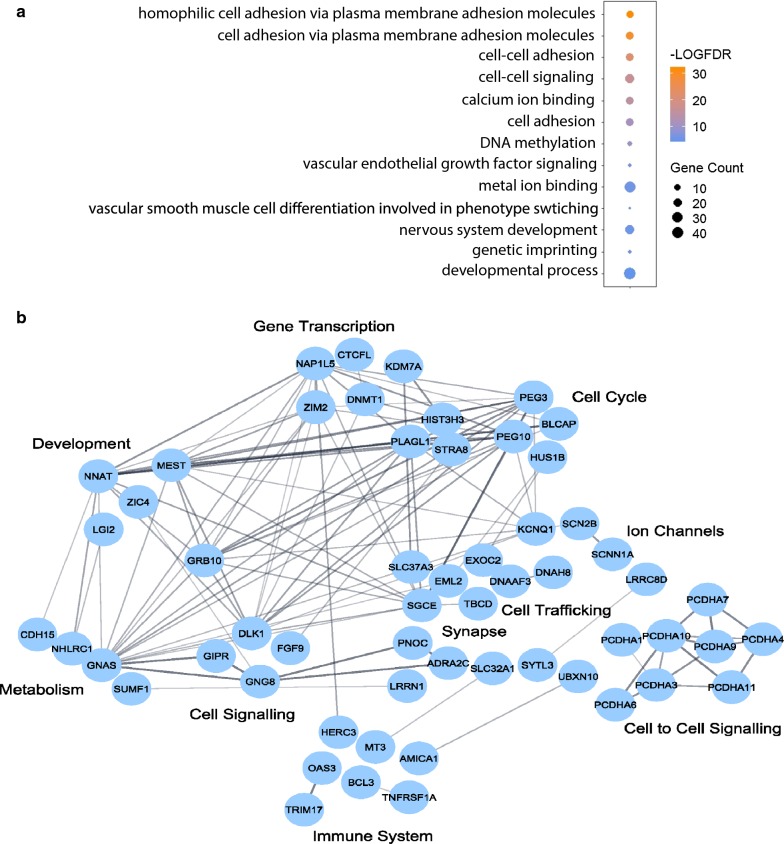
Functional enrichment analysis of DNMT1-dependent genes conserved in the human genome. **a** Gene Ontology enrichment for cellular processes and molecular functions from the 112 selected human DNMT1-dependent genes (adjusted *p* value; false-discovery rate FDR < 0.05). **b** Network interactions from STRING database with an interaction enrichment *p* value < 1 × 10^−16^. Genes are clustered based on functional gene annotations from reactome pathways

Out of the 145 regions identified in the human genome, we selected 97 of the most highly conserved regions compared to the mouse genome. The genes nearest to these regions on the same allele (112 genes) were used for functional enrichment analyses (Table 1). We used Gene Ontology functional annotations to gain insights into the cellular processes associated with these genes and significance was determined from the Fisher’s exact test with *p* value adjustment using false-discovery rate method (FDR < 0.05). We observed adjacent genes were highly associated with cell to cell interactions and signaling. The number of genes involved in this biological process as well as the significance of its enrichment (expressed as log base 2 false-discovery rate) are shown in Fig. 2a. Genes regulating cell–cell adhesion belonged primarily to the cadherin protein family. This agrees with previous reports showing monoallelic expression of protocadherins in Purkinje neurons [19]. Gene Ontology of cellular components describes the subcellular compartments where enriched cellular processes and molecular functions occur. The plasma membrane and the pre-synapse were significantly enriched in our dataset in accordance with the enrichment of cell–cell interactions and calcium ion binding (FDR < 0.05, Additional file 1: Table S3).

**Table 1:** Predicted human DNMT1-dependent loci and genes

chr1:203012544–203013111	*PPFIA4*
chr9:130633077–130634324	*AK1*
chr20:2736325–2736555	*EBF4*
chr20:30133018–30134961	*MCTS2P/HM13*
chr20:30134986–30135324	*MCTS2P/HM13*
chr20:36149063–36149960	*NNAT/BLCAP*
chr20:36150645–36151532	*NNAT/BLCAP*
chr20:37356947–37357083	*SLC32A1*
chr20:57424303–57428479	*GNAS*
chr20:57429454–57430628	*GNAS*
chr20:57428494–57429364	*GNAS*
chr20:57430647–57430802	*GNAS*
chr1:151762641–151762866	*TDRKH*
chr1:40769328–40769695	*COL9A2*
chr1:23789986–23792384	*ASAP3*
chr1:20569708–20569903	*UBXN10/VWA5B1/LINC01757*
chr1:17570695–17571208	*PADI1*
chr4:3809685–3810214	*ADRA2C*
chr4:6575638–6576768	*MAN2B2*
chr4:24982328–24982857	*CCDC149/LGI2*
chr1:90308879–90309031	*LRRC8D*
chr1:90309136–90309398	*LRRC8D*
chr12:120031502–120031941	*TMEM233*
chr12:117146822–117147291	*C12orf49*
chr12:113400413–113400788	*OAS3*
chr7:94284623–94285630	*SGCE*
chr7:94285746–94285993	*PEG10*
chr7:94286061–94286219	*PEG10*
chr7:94286263–94287973	*PEG10*
chr7:130126204–130127042	*MEST*
chr7:130129122–130129865	*MEST*
chr7:130130320–130132940	*MEST*
chr7:130133202–130135402	*MEST*
chr7:134955283–134955477	*STRA8*
chr7:139942121–139942907	*KDM7A/SLC37A3*
chr4:89618037–89618988	*NAP1L5/HERC3*
chr3:3842722–3843091	*LRRN1/SUMF1*
chr12:6451268–6451802	*TNFRSF1A/SCNN1A*
chr12:14518500–14518687	*ATF7IP*
chr19:55677470–55677864	*DNAAF3*
chr19:57349655–57353647	*PEG3/ZIM2/MIMT1*
chr19:47138150–47139513	*GNG8*
chr19:46148498–46149609	*EML2/GIPR*
chr19:45260696–45260939	*BCL3*
chr19:44008046–44008221	*PHLDB3*
chr19:42810915–42811210	*PRR19*
chr11:2018645–2019501	*H19*
chr11:2718814–2720223	*KCNQ1OT1/KCNQ1*
chr11:2720461–2722038	*KCNQ1OT1/KCNQ1*
chr4:175134957–175135614	*AK125257/FBXO8*
chr16:56624355–56625029	*MT3*
chr16:67313281–67313411	*PLEKHG4*
chr16:72698159–72698950	*AK201563/LINC01572*
chr16:81297176–81297683	*BCMO1*
chr16:89258400–89259894	*CDH15*
chr12:1100277–1100455	*ERC1*
chr19:10250933–10251636	*DNMT1*
chr11:118050777–118051248	*SNC2B/AMICA1*
chr15:79574796–79575831	*ANKRD34C*
chr3:147111981–147112692	*ZIC4*
chr6:144328582–144329817	*PLAGL1*
chr19:616132–616452	*HCN2*
chr7:50850069–50850216	*GRB10*
chr2:63271636–63272171	*EHBP1*
chr1:228612850–228612928	*HIST3H3/TRIM17*
chr17:10551758–10552335	*MYH3*
chr17:75723668–75724123	*LINC01987*
chr17:76133291–76133628	*TMC8*
chr17:80187539–80188383	*SLC16A3*
chr17:80797978–80799678	*TBCD*
chr14:74814669–74815870	*VRTN*
chr14:101275797–101278087	*MEG3/DLK1/MIR2392*
chr14:101290612–101291411	*MEG3*
chr14:101292483–101292929	*MEG3*
chr14:101292955–101294422	*MEG3*
chr6:656305–657021	*HUS1B/EXOC2*
chr6:18121790–18122423	*NHLRC1*
chr9:94123015–94123883	*AUH*
chr13:22246327–22246913	*FGF9*
chr8:28196773–28197118	*PNOC*
chr8:24771898–24773551	*NEFM*
chr8:24771655–24771790	*NEFM*
chr13:53423771–53424148	*PCDH8*
chr3:185795915–185797093	*ETV5*
chr6:158422346–158423188	*SYNJ2*
chr6:159084156–159084536	*SYTL3*
chr5:172306505–172306903	*ERGIC1*
chr6:36237727–36237965	*PNPLA1*
chr6:38997730–38998017	*DNAH8*
chr2:43058554–43059906	*HAAO*
chr4:11280093–11280328	*MIR572/HSTS31*
chr18:34823790–34823894	*CELF4*
chr5:140175896–140176159	*PCDHA2/PCDHA1*
chr5:140228130–140228418	*PCDHA1*-*9*
chr5:140250568–140251167	*PDDHA1*-*11*
chr5:140554155–140554621	*PCDHB7*
chr18:56664106–56665166	*ZNF532/OACYLP*

The interaction of the proteins encoded by DNMT1-dependent genes was analyzed using the STRING database (Fig. 2b). The STRING database is a commonly used platform that summarizes the functional associations of a group of proteins. Out of the 112 selected protein-encoding genes in humans, 98 nodes with 109 edges were detected with a medium confidence interaction score (> 0.4). The interaction *p* value (PPI) was less than 1 × 10^−16^, indicating that the number of associations was significant. We manually clustered genes with interactions using functional gene annotations. The largest cluster consisted of genes involved in cell–cell signaling including cadherins and cell surface adhesion molecules, cell trafficking chaperones, cytoskeleton proteins, and voltage-gated ion channels. Other significant pathways included developmental pathways of the nervous system and the vascular system.

### Human DNMT1-dependent genes are enriched in tissues of the brain

**Fig. 3:**
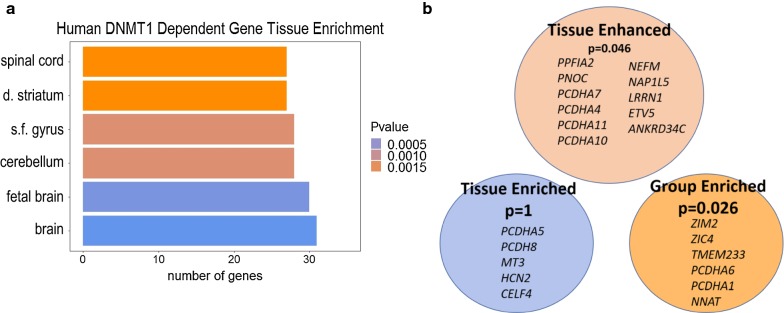
Tissue enrichment analysis of DNMT1-dependent genes conserved in the human genome. **a** The top 6 tissues represented from the 112 selected human DNMT1-dependent genes (adjusted *p* value < 0.01) scored from Enrich R using the ArchS4 human tissue database (*s.f. gyrus* superior frontal gyrus, *d. striatum* dorsal striatum). **b** DNMT1 human genes that are tissue-enriched, tissue-enhanced, or group-enriched genes within the cerebral cortex from Tissue Enrich (adjusted *p* value shown). TissueEnrich definitions in “Methods” section

Given the evidence that DNMT1-dependent genes may play an important role in cell–cell adherence and communication as well as in nervous system development, we hypothesized that DNMT1-dependent genes may be highly expressed in the brain. We analyzed the enrichment of tissue expression using the ARCHS4 human tissue database in EnrichR. The ARCHS4 database reports publicly available RNA-sequencing across all tissues and cell types in approximately 85,000 human samples [41]. Significant expression of the DNMT1-dependent genes in the adult and developing brain was observed (*p* < 0.01, Fig. 3a). We also observed enrichment in the regions of the brain associated with motor function control. These structures include the cerebellum, spinal cord, and the striatum which are directly involved with motor coordination as well as the superior frontal gyrus which contains the supplementary motor area activated in complex movements [43, 67].

To further determine if these genes were specific to neuronal tissues or if they have functionality across several tissues, we analyzed DNMT1-dependent genes for tissue-specific enrichment using the TissueEnrich R package [31]. Genes with increased expression in one tissue compared to the expression in any other tissue were defined as tissue enriched while genes with increased expression in one tissue compared to the average of all tissues were defined as tissue enhanced. Group-enriched genes were defined as genes that have increased expression in a group of tissues compared to all other tissues. Our analysis demonstrates that DNMT1-dependent genes conserved in the human genome have a significant abundance of tissue enhanced and group-enriched genes within the brain, but not tissue-enriched genes (Fig. 3b). From these data, we conclude that DNMT1-dependent genes are likely important in cellular processes in the fetal and adult brain.

### Five DNMT1-dependent genes are represented in genes for potential PD blood biomarkers in patients

To further explore the significance of identified human DNMT1-dependent genes, we evaluated the recent literature on potential blood biomarkers in Parkinson’s disease (PD) patients. Encouragingly, we observed five differentially methylated genes in these studies within our conserved human regions [29, 75]. They are *COL9A2*, *SCNN1A*, *AMICA1*, *SLC16A3*, and *DLK1*. One of these genes, *COL9A2*, was also found to be differentially expressed in our rotenone treated cells [29] (described below). Given that candidate PD biomarkers and our DNMT1-dependent genes were selected by differing criteria, we consider five overlapping genomic regions to be promising toward our hypothesis.

### Human DNMT1-dependent genes are differentially expressed in response to rotenone in human cells

We have shown that DNMT1-dependent regions have conserved sequences in the human genome and are enriched at genes involved in cell to cell interactions (Fig. 2). These genes have enhanced expression in the brain and may contribute to neurological dysfunction and disease in response to environmental stress (Fig. 3). To test the hypothesized contribution, we focus on rotenone exposure. Rotenone is a mitochondrial complex I inhibitor that is known to disrupt neuronal cell function in Parkinson’s disease-associated brain regions [72]. These brain regions include the cerebellum, spinal cord, striatum, and basal ganglia. We treated human cell line HEK293 with rotenone (200 nm) for 24 h. This dose was chosen based on previous reports in HEK293 and other neuronal cell models [28, 56, 73]. Rotenone treatment had a substantial effect on the expression levels of over 2000 genes (≥ 1.5-fold, FDR ≤ 0.05). We examined these differentially expressed genes with our identified human DNMT1-dependent genes and discovered 14 of them had been changed upon rotenone treatment (Table 2). We validated the expression of 7 of these genes with qRT-PCR (*R*^2^ = 0.69, Additional file 1: Figure S2).

**Table 2:** Human DNMT1-dependent genes altered by rotenone

Gene	Log2FC	FDR	Region type	Function/process
*MYH3*	− 1.62	3.31E−09	Intragenic	Myosin protein; cell movement and transport
*PPFIA4*	− 1.37	2.59E−13	Intragenic	Neurotransmitter release synaptic function
*COL9A2*	− 0.79	1.53E−04	Intragenic	Collagen; extracellular matrix organization
*DNAAF3*	− 0.78	3.59E−02	Intragenic	Dynein protein assembly; cell movement
*LRRC8D*	− 0.78	8.42E−05	Intragenic	Ion channel protein; neurotransmission
*PLEKHG4*	− 0.65	2.74E−03	Intragenic	Guanine exchange factor; cell signaling
*ADRA2C*	0.59	2.93E−02	Intergenic	Neurotransmitter release synaptic function
*EML2*	0.62	5.99E−05	Promoter	Microtubule protein; synaptic function
*HCN2*	0.66	1.10E−03	Intragenic	Voltage gated ion channel; action potential
*KDM7A*	0.67	1.16E−04	Intergenic	Histone demethylase; neurodevelopment
*PHLDB3*	0.76	3.75E−04	Intragenic	Enzyme binding; cell growth and proliferation
*GIPR*	0.76	2.37E−02	Promoter	Gastric inhibitory peptide; insulin release
*BCL3*	0.78	2.00E−02	Intragenic	Proto-oncogene; cell growth and proliferation
*NEFM*	0.80	4.33E−07	Intragenic	Neurofilament; synaptic function

Log2FC is the log base twofold change of the fragments per kilobase of exon per million fragments mapped (FPKM) or “read counts” of the rotenone-treated *HEK293* relative to the vehicle control. FDR is the adjusted *p* value using the false-discovery rate correction for multiple hypotheses

**Fig. 4:**
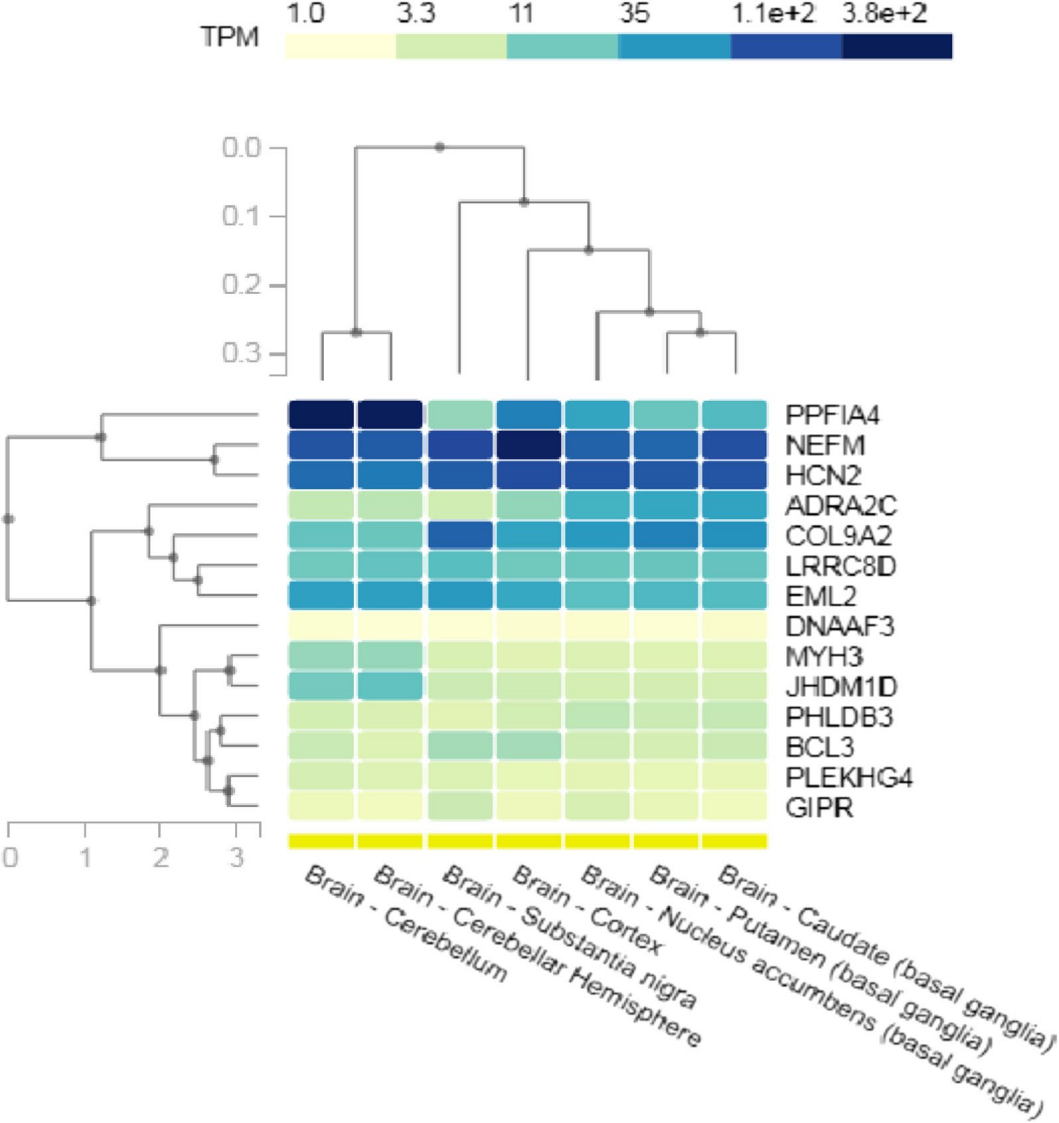
Regional expression of DNMT1-dependent genes altered by rotenone. Human DNMT1-dependent genes that were differentially expressed (≥ 1.5-fold change, false-discovery rate FDR ≤ 0.05) in response to rotenone were used for regional expression analysis in the Genotype-Tissue Expression (GTex) database [46]. Brain regions selected are associated with Parkinson’s disease pathogenesis and important for motor function control. The heat map was generated using GTex and organization is clustered by gene function and tissue function. The color of each square indicates the level of expression of the gene in the selected region in transcripts per kilobase million (TPM) represented by the color bar (dark blue = higher expression)

We investigated whether these genes may contribute to rotenone-induced Parkinson’s disease by observing their expression in Parkinson’s disease tissues (Fig. 4). All 14 genes were expressed in Parkinson’s disease regions (> 1 TPM) and 8 of the genes (*PPFIA4*, *NEFM*, *HCN2*, *ADRA2C*, *COL9A2*, *LRRC8D*, *EML2*, and *KDM7A*/*JHDM1D*) had pronounced expression in Parkinson’s disease regions (> 35 TPM). Two genes, *NEFM* and *HCN2*, had significant expression (> 100 TPM) in all selected regions and were identified by our tissue-specific enrichment analysis as tissue enhanced and tissue enriched, respectively (Fig. 3b). Notably, *Hcn2* was investigated favorably for having a switchable allele-specific methylated phenotype in our mouse model [48]. We therefore selected *NEFM* and *HCN2* for targeted methylation analysis based on their regional expression and significant up-regulation from both RNAseq and qRT-PCR analyses. The relevance and significance of *HCN2* and *NEFM* in human development and diseases are detailed later in “Discussion” section.

### DNMT1-dependent regions at *HCN2* and *NEFM* are differentially methylated in response to rotenone in human cells

**Fig. 5:**
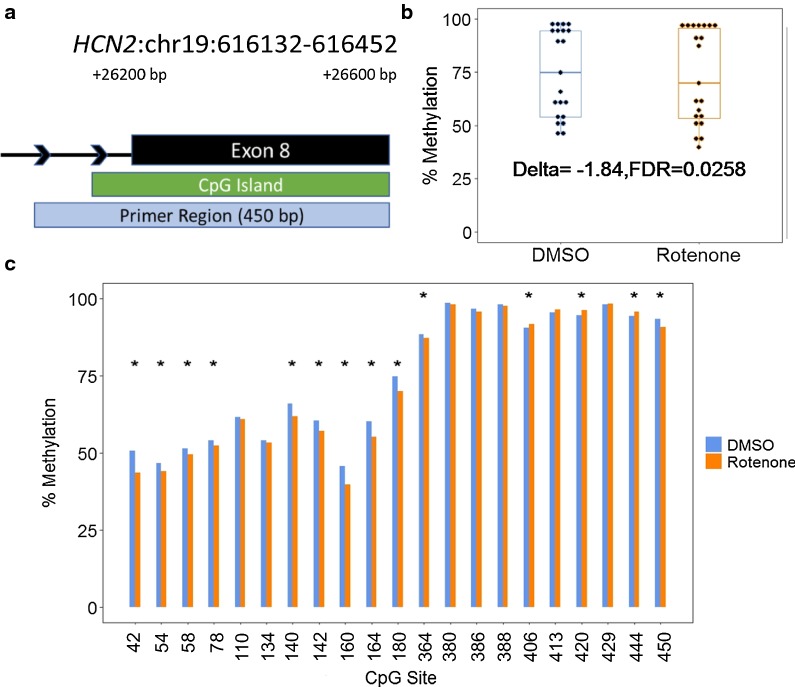
Altered CpG methylation at *HCN2* human DNMT1-dependent locus. **a** Genomic location of identified *HCN2* DNMT1-dependent region. The DNA element and distance from the transcription start site are annotated in black. The primer region box indicates the amplified region for Bisulfite-sequencing. **b** The percent methylation of all CpG sites within the amplified region. Delta indicates the change in the mean CpG methylation percentage and the associated false-discovery rate. **c** The percent methylation of individual CpG sites within the amplified region. Significant differentially methylated cytosines are indicated by * (Δ > 1%; *q* value < 0.01)

Previously, germline ASMs are especially vulnerable to environmental exposure, thereby altering imprinted gene expression [70]. Herein, we determined the potential methylation changes of the defined DNMT1-dependent region at these two genes, *NEFM* and *HCN2*, with significant up-regulation in response to rotenone. We completed base-resolution bisulfite sequencing of these regions amplified with bisulfite PCR. After filtering of low-quality reads, approximately 42% of reads were mapped uniquely to the amplified regions. We observed high correlation between biological replicates and similar average coverage between control and treated samples (Additional file 1: Figures S2–S3). The average CpG coverage for both genes in all samples was > 15,000×. Of the 23 predicted CpG sites within the amplified DNMT1-dependent region on exon 8 of *HCN2*, 21 CpGs had adequate coverage (> 1000×) in all samples and 14 CpGs were differentially methylated (Fig. 5; Additional file 1: Table S4). These differentially methylated cytosines were largely hypomethylated (Additional file 1: Figure S4). The mean percent methylation of all CpGs within the amplified DNMT1-dependent region was also significantly hypomethylated (Δme of − 1.84%, FDR < 0.05).

**Fig. 6:**
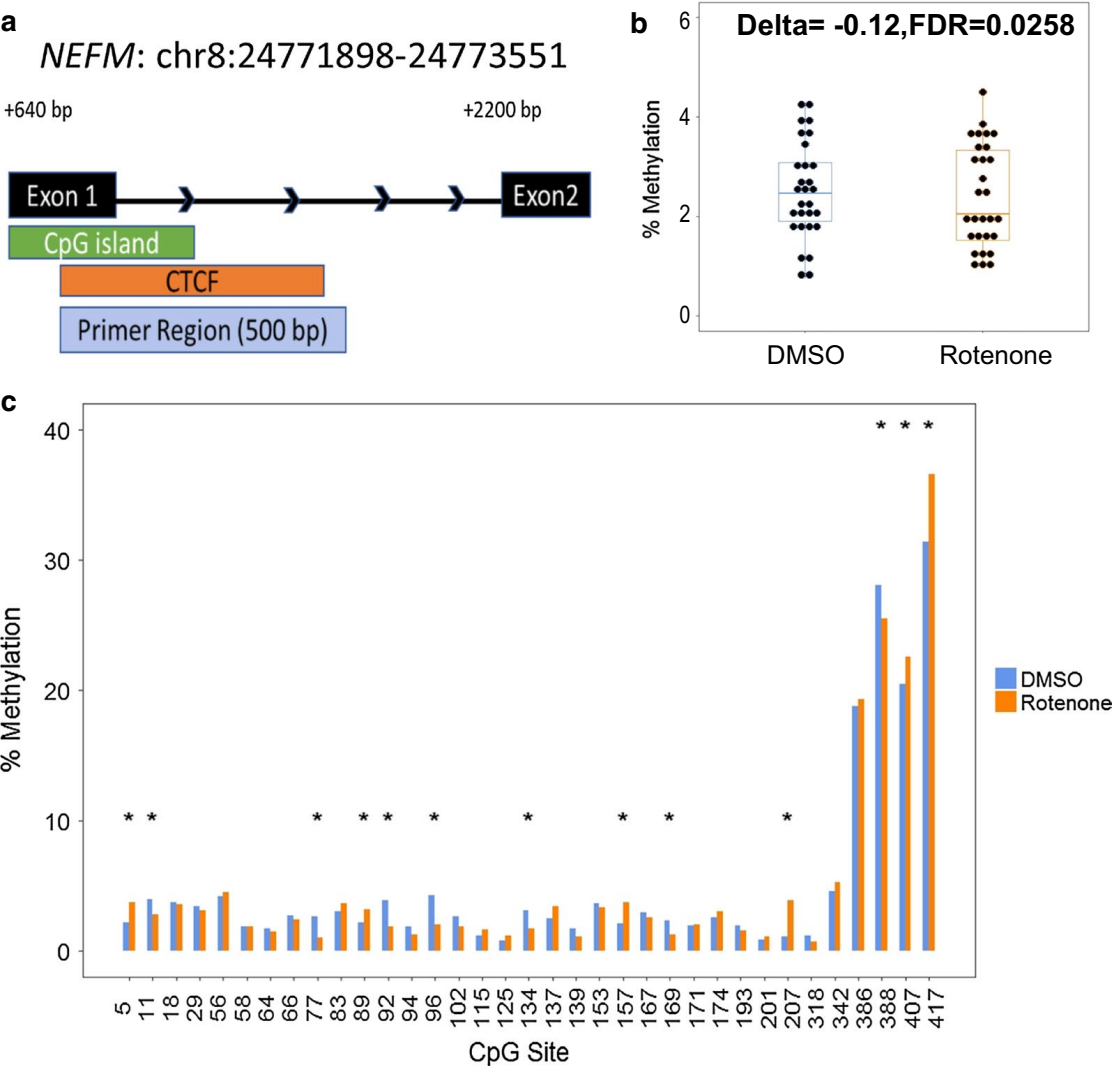
Altered CpG methylation at *NEFM* human DNMT1-dependent locus. **a** Genomic location of the identified *NEFM* DNMT1-dependent region. The DNA element and distance from the transcription start site are annotated in black. The transcription factor-binding site for CTCF was annotated from ENCODE v2 and ENCODE Uniform TFBS tracks in Genome Browser. The primer region box indicates the amplified region for Bisulfite-sequencing. **b** The percent methylation of all CpG sites within the first 200 base-pairs of the amplified region. Delta indicates the change in the mean CpG methylation percentage and the associated false-discovery rate. **c** The percent methylation of individual CpG sites within the amplified region. Significant differentially methylated cytosines are indicated by * (Δ > 1%; *q* value < 0.01)

We saw a similar trend in the DNMT1-dependent region at exon 1 of *NEFM*. Of the 39 predicted CpG sites within the amplified DNMT1-dependent region, 35 CpGs had adequate coverage in all samples and 13 of these CpGs were differentially methylated. A slight majority (54%) of these differentially methylated cytosines were hypomethylated (Additional file 1: Figure S4). The overall change in methylation ratio for the entire region was significant but very low (< 0.1% absolute difference, FDR < 0.01). As a result, we decided to focus on the first 200 bp of the 500-bp amplified region, which overlap both the CpG island at exon 1 as well as a CTCF transcription factor binding site reported by ENCODE [18]. In this region, there was a slightly higher change in methylation (Δme of − 0.12%, FDR < 0.05). In addition, we used the CTCF binding site prediction tool to determine the exact CpG sites within the CTCF binding motif [84]. One of the top hits predicted CpG binding on the negative strand at CpG sites 89–96 within the defined *NEFM* region (Fig. 6; Additional file 1: Table S5). Three of these four CpG sites were differentially methylated with half of them having > 2% reduced methylation.

These data enable us to conclude that the methylation status of DNMT1-dependent regions in the human genome is vulnerable to the neurotoxicant rotenone. We found that the coding regions and transcription factor binding motifs may be among the DNA elements that are particularly susceptible to exposure. The changes in methylation we observed were similar in scale to observed differential methylation at gene-encoding regions in the blood and brain of Parkinson’s disease patients [29, 49, 52, 75]. Both *HCN2* and *NEFM* are regionally expressed in Parkinson’s disease tissues and their function has a critical role in neuronal plasticity and survival detailed in “Discussion” section.

## Discussion

Our previous work identified DNMT1-dependent putative non-germline ASMs in the mouse genome. In this study, we analyzed these regions and found that 70% were in highly conserved regions with the human genome. In the human genome, our candidate loci were often located at gene-coding regions and half of them overlapped transcription factor binding sites (Fig. 1). Our observations agreed with another recent study which identified genome-wide ASMs in human samples from a Norfolk Island genetic isolate [8]. Methylated cytosines alter gene expression by influencing the binding of transcription factors to DNA. We listed the top transcription factor binding sites within our identified candidate regions in human embryonic stem cells using ENCODE experimental data (Fig. 1). Unsurprisingly, three of these transcription factors were TAF1, TBP, and POL2RA which all have an essential role in initializing transcription. We were interested to see SIN3A and RBBP5 which both interact with histone-modifying enzymes to regulate chromatin accessibility and are critical during neurodevelopment [24]. Furthermore, SIN3A is recruited to the methyl-CpG binding protein MeCP2 to silence transcription. Mutations in MeCP2 cause an X-linked neurodevelopmental disorder known as Rett Syndrome and similarly impairment of SIN3A expression also causes developmental cognitive deficits [78]. MeCP2 and SIN3A have been linked to the establishment and maintenance of imprinting control regions but their effect on the expression of neighboring imprinted genes remains to be determined [47].

CTCF is another transcription factor of interest with 17% of the DNMT1-dependent human regions overlapping the CCCTC-binding motif. CTCF is also critical in neurodevelopment and chromatin organization [14, 21]. CTCF mediates the formation of chromatin loops and thus can promote widespread changes in gene expression [30, 58]. When bound to sequences known as insulator sequences, CTCF represses transcription by blockading promoter–enhancer interactions [7, 27]. CTCF and the stabilizing protein cohesion bind at numerous imprinted control regions [60, 65]. CTCF has been reported to preferentially bind unmethylated chromatin but binding affinity depends not only on the methylation status of the motif itself but the surrounding CpG sites as well [77, 81].

Given the importance of imprinted gene clusters in development, we analyzed functional enrichment of our non-germline ASMs in the human genome. The most significant biological process associated with our gene set was cell to cell adhesion (Fig. 2). We observed a significant group of cadherins at DNMT1-dependent regions on chromosome 5. Cadherin proteins are expressed on the membrane of embryonic stem cells and are critical for their self-renewal by forming tight intracellular niches [59]. The expression of cadherin subtypes on embryonic stem cells is variable and the patterning of cadherin expression also controls their differentiation. Protocadherins are involved in neuronal connectivity and this function extends from neural progenitors during development into postmitotic neurons in the adult brain [66]. Intriguingly, protocadherin is regulated by CTCF and deletion of CTCF in mice caused deficits in hippocampal learning and memory via dysregulation of protocadherin expression [66]. The most significant molecular function was calcium-ion binding and the pre-synaptic axon terminal was one of two most significant cellular components represented. This agreed with our network analyses where multiple genes were involved in cell trafficking and synaptic activity (Fig. 2). We investigated whether developmental genes were specific to an individual tissue or group of tissues. These genes from DNMT1-dependent regions have significant enrichment of genes expressed in the cerebral cortex from two separate databases, EnrichR ArchS4 and Tissue Enrich Human Protein Atlas (Fig. 3). Our data suggest that DNMT1-dependent non-germline ASMs have enhanced expression in the brain, which could be important for neurological development and cellular communication function.

In our previous work, we characterized non-germline ASMs in the mouse genome at two genes, *Hcn2* and *Park7*, with potential in Parkinson’s disease [9, 38, 48]. The proper maintenance of the epigenome throughout aging is believed to have a major impact on the risk of neurodegeneration later in life [25, 40]. The influence of germline ASMs on neurodegeneration has recently been of interest in the literature given their involvement in neurodevelopment but the effect of non-imprinted ASMs has not been well characterized [25]. To experimentally examine the association of identified ASMs in the human genome with Parkinson’s disease, we used human embryonic kidney cells with a neuronal lineage phenotype and treated them with rotenone for 24 h [69]. We observed several of our candidate genes were affected in response to rotenone treatment and half of these genes have regional expression in Parkinson’s disease-associated regions (Fig. 4). Among these genes, *HCN2* and *NEFM* were determined from our tissue enrichment analysis to have a higher expression level in the brain than any other tissue. In addition, experimental analysis of *Hcn2* in the mouse genome suggests a random, switchable allele-specific methylation pattern that was independent of the parent-of-origin [48]. We selected these two genes for methylation analysis to determine if conserved non-germline ASMs in the human genome were sensitive to environmental factors associated with Parkinson’s disease.

The *HCN2* gene encodes an isoform of the hyperpolarization-activated cyclic nucleotide-gated channel located on the membrane of neurons in the central and peripheral nervous system. HCN channels regulate neuronal plasticity and have the advantage of using both voltage dependent mechanisms as well as cAMP intracellular signaling mechanisms [16]. In the midbrain, these channels control the spontaneous activity of dopaminergic neurons and their dysfunction has been linked to the depletion of dopamine in Parkinson’s disease [13, 16, 26]. In the human genome (hg19), the conserved DNMT1-dependent locus identified was 321 bp at a CpG island on exon 8 of the gene. We observed significant upregulation of mRNA expression levels (1.6-fold change, FDR < 0.01) that correlated with DNA hypomethylation (− 1.8%, FDR < 0.05) of a 450-bp site surrounding the region of interest (Table 2; Fig. 5; Additional file 1: Table S4). Dysregulated *HCN2* expression could affect HCN2 channel activity leading to disrupted regulation of dopaminergic excitability.

The *NEFM* gene encodes a subunit of neuron-specific intermediate filaments known as neurofilaments. Neurofilaments are primary components of myelinated axons and are essential for synaptic function [80]. Neurofilament subunit expression is tightly regulated to maintain proper stoichiometry. As such, aberrant expression of *NEFM* likely disrupts axonal growth and transport. Interestingly, neurofilament subunits including the NEFM protein are considered promising neurodegeneration biomarkers due to their cell specificity and sensitivity to neuronal damage [36]. In Parkinson’s disease patients, neurofilament proteins have been detected at higher levels in the cerebral spinal fluid, and more recently, in the blood [1, 63, 64]. In our data, the conserved DNMT1-dependent locus covered a 150-bp region in exon 1 as well as a 1650-bp region spanning exon 1 to intron 2. We observed significant upregulation of mRNA (1.7-fold change, FDR < 0.01) and hypomethylation of a 200-bp section of the identified region at exon 1 (− 0.12%, FDR < 0.05). While the total change in percent methylation was relatively small, the selected region contained a CTCF-binding site. Several CpG sites located within this CTCF-binding motif had higher changes in methylation (> 1%, adjusted *q* value < 0.01) (Fig. 6; Additional file 1: Table S5). As mentioned previously, CTCF is hypersensitive to changes in DNA methylation and approximately 41% of CTCF-binding variability has been attributed to DNA methylation [77]. The lack of repressive signaling from CTCF could contribute to the observed increases in NEFM reported in Parkinson’s disease patients and the observed overexpression of *NEFM* associated with cytoplasmic inclusions in motor-impaired mice [45, 68, 79].

We evaluated the recent literature on candidate blood biomarkers in Parkinson’s disease patients and observed five differentially methylated genes (*COL9A2*, *SCNN1A*, *AMICA1*, *SLC16A3*, and *DLK1*) in these studies within our conserved human regions [29, 75]. Of these genes, *COL9A2* was differentially expressed in our rotenone treated cells and was determined to have high regional expression in the substantia nigra (Table 2, Fig. 4) [29]. This observation is strengthened with another study that has found that differentially methylated genes in the blood have high concordance with differentially methylated genes in the brain [49]. These data partially support our hypothesis that environmentally induced changes in DNMT1-dependent ASMs in the human brain can alter the risk of neurodegeneration.

## Conclusions

DNMT1 expression in neural stem cells is essential for adult neurogenesis and the survival of adult neurons in the brain [54]. We have shown that non-germline ASMs are dependent on DNMT1 in mice. The goal of this study was to identify conserved DNMT1-dependent regions and putative non-germline ASMs in the human genome and test the vulnerability of these regions to a neurotoxicant associated with Parkinson’s disease. Our work identified candidate, non-germline ASMs with DNMT1 dependence as enriched in the human brain. We discovered 14 genes have altered expression (> 1.5-fold change) at predicted ASMs in response to rotenone. We quantified methylation of 2 identified regions at adjacent genes (*HCN2* and *NEFM*) known to increase the risk for Parkinson’s disease and observed significant hypomethylation. In the future, a larger panel of these identified regions in the human genome will be tested in other cells’ lines at varying points in neuronal differentiation to determine the role of non-germline ASMs on neuronal development and its maintenance with age.

## Methods

### Identification of conserved DNMT1-dependent regions in the human genome

Whole Genome Bisulfite Sequencing (WGBS) was used to analyze base resolution methylomes of a series of *Dnmt1* (−/−), *Dnmt3a* (−/−), and *Dnmt3b* (−/−) murine embryonic stem cell lines (wild-type J1) as described previously [44, 48]. DNMT1-dependent regions termed “NORED” were defined as regions with near complete loss of methylation in *Dnmt*1 (−/−) compared to wild-type J1 that remained unable to recover methylation after the addition of exogenous *Dnmt1* cDNA. To identify the conserved DNMT1-dependent regions in the human genome, we used the UCSC Genome Browser LiftOver software to locate regions in the hg19 assembly from the mouse mm10 assembly. A text file of the chromosome positions (chr: start–end) for each putative ASM was uploaded into LiftOver and converted to the human hg19 assembly with a minimum ratio of 0.1 bases mapping for each region. The genomic location of each conserved region was analyzed in the UCSC Genome Browser window with NCBI RefSeq annotations. Transcription factor binding was observed using the Uniform Transcription Factor Binding data found in the ENCODE Regulation super track. We selected all transcription factor binding sites in H1-human embryonic stem cells (H1-hESCs) detected with CHIP-Seq experiments from the ENCODE consortium from 2007 to 2012 [18]. Imprinted genes from mouse and human genome were identified from the Jirtle Laboratory GeneImprint database (http://www.geneimprint.org/).

### Functional enrichment for candidate DNMT1-dependent genes in the human genome

We restricted functional enrichment analysis of conserved human regions to those that had > 70% base pair match for over 90% of the span of the identified region (Table 1). Pathway enrichment and network interactions for human genes nearest to these regions were calculated using the STRING database [71]. Gene Ontology was used for functional annotations and significance was measured using Fisher’s exact test with a false-discovery rate (FDR) < 0.05. Network interactions were clustered using Cytoscape based on gene functional annotations in the reactome pathways [20].

### Tissue enrichment for candidate DNMT1-dependent genes in the human genome

The human DNMT1-dependent genes (Table 1) were used for tissue enrichment in EnrichR [12] using the ArchS4 database [41]. The top six human tissues were reported with a *p* value < 0.01 adjusted using their correction for the Fisher’s exact test [12]. Enrichment of tissue-specific genes was performed using the TissueEnrich R package which uses gene expression data from the Human Protein Atlas [31]. Genes with an expression level of at least one transcript per million (TPM) were defined as tissue enriched when expression was at least fivefold higher in a distinct tissue compared to the expression of any other individual tissue and tissue enhanced when expression was at least fivefold higher in a distinct tissue compared to the total average expression of all other tissues. Group-enriched genes were defined as genes with an expression level of at least one TPM and had at least fivefold higher expression in a group of tissues compared to all tissues. These definitions were taken from TissueEnrich.

### Cell culture and treatment of human cell line HEK293

All media reagents and chemicals in cell culture were purchased from Sigma (St. Louis, MO). Human cell line HEK293 was grown in Dulbecco’s Modified Eagle Medium with high glucose, l-glutamine, and sodium pyruvate. Media were supplemented with 10% (v/v) heat inactivated fetal bovine serum and 1% (v/v) Penicillin–Streptomycin. HEK293 cells were confirmed by ATCC. Cells were treated at approximately 70% confluency with 200 nM rotenone or DMSO vehicle control (< 0.001%) for 24 h. Cell viability was measured with trypan blue (0.4%) staining. Viable and dead cells were counted manually using a hematocytometer. A minimum cell viability of 85% was used for all experiments.

### RNA extraction and RNA sequencing library construction

Total RNA was extracted from two replicates of DMSO or rotenone-treated HEK293 using the trizol method (Invitrogen, Carlsbad, CA). A total of 2 μg per sample was used for library construction using the TruSeq Sample Preparation kit from Ilumina (San Diego, CA). Poly-A containing mRNA molecules were isolated from total RNA using oligo-dT attached magnetic beads. Isolated mRNA was then fragmented and synthesized into double-stranded cDNA according to the kit instructions. Ligation of unique Ilumina adapter indices was completed for each sample before bead purification. Libraries were loaded onto a 2% agarose gel and library products between 200 and 800 bp were purified using the mini-Elute gel extraction kit from Qiagen (Hilden, Germany). Approximately 150 ng was sent for sequencing on a HiSeq2000 platform with 100 bp paired-end reads.

### RNA sequencing data analysis

Adapter sequences were removed from the raw sequencing data and individual libraries were converted to the fastq format. Sequencing reads were aligned to the human genome (hg19) with TopHat2 (v2.0.9) [37]. For mRNA analyses, the RefSeq database (Build 37.3) was chosen as the annotation references. Read counts of annotated genes were obtained by the Python software HTSeq-count [4]. The read counts of each transcript were normalized to the length of the individual transcript and to the total mapped fragment counts in each sample and expressed as fragments per kilobase of exon per million fragments mapped (FPKM) of mRNAs in each sample. Differentially expressed genes were defined as those with a 1.5-fold change in expression using a false-discovery rate (FDR) *p* value adjustment < 0.05 from the edgeR package [62]. We examined differentially expressed genes in RNA-seq in common with the genes nearest to the conserved DNMT1-dependent regions in the human genome with > 70% base-pair match for over 90% of the span of the identified region in the mouse genome. Overlapping genes were entered into the Genotype-Tissue Expression (GTEx) database using the multi-gene query tool [46]. Parkinson’s disease brain regions associated with motor function including the cerebellum, cortex, frontal cortex, spinal cord, substantia nigra, and basal ganglia were selected for further expression analysis.

### RNA sequencing validation with quantitative reverse-transcription PCR

Total RNA was extracted from an additional replicate of HEK293 treated with DMSO or rotenone using the same procedure as stated above. A total of 500 ng RNA was converted to cDNA with the PrimeScript RT reagent kit with gDNA eraser from Takara (Kusatsu, Japan). We selected 7 out of 14 overlapping genes for quantitative PCR (qRT-PCR) analysis using primers listed in Additional file 1: Table S1. All qRT-PCRs were performed on a 7500 Real-Time PCR system from Applied Biosystems (Foster City, CA) using the iTaq Universal SYBR Green Supermix from Bio-Rad (Hercules, CA). The change in expression was normalized to the GAPDH housekeeping gene and expressed as fold change (2-^ΔΔCT^).

### Bisulfite-DNA conversion and Bisulfite-amplicon sequencing library construction

Genomic DNA was extracted from two replicates of DMSO or rotenone-treated HEK293 using Phenol:Chloroform:Isoamyl alcohol (Sigma, St. Louis, MO). A total of 200 ng DNA was Bisulfite-converted using the Sigma DNA Imprint Modification kit two-step protocol. Bisulfite-converted DNA (BS-DNA) was amplified with primers for selected DNMT1-dependent regions designed with MethPrimer [42] (Additional file 1: Table S2). Amplified BS-DNA products were run on a 2% EtBr agarose gel and purified using the mini-Elute gel extraction kit from Qiagen (Hilden, Germany). Purified products for each sample were pooled together and 1 ng was used for library preparation using the Ilumina Nextera DNA Library Preparation kit. Each sample was tagged with a unique Nextera XT adapter (San Diego, CA). Sequencing libraries were quality checked via Bioanalyzer and run on an Ilumina MiSeq platform to generate 150 bp paired end reads.

### Bisulfite-amplicon sequencing analysis

The raw fastq files were imported into the Galaxy web platform [2]. Reads with quality score > 30 were trimmed with Trim Galore [39]. Reads were mapped to amplified sequences in the human genome (hg19) using bwa-meth [57]. MethylDackel was used for methylation calling and per-cytosine contexts were merged into per-CPG metrics (https://github.com/dpryan79/MethylDackel). Duplicates and singletons identified in alignment were ignored from the methylation call. Minimum and maximum per-base depths were 1000× and 100,000×, respectively. The output was selected for methylKit format. Coverage statistics and differentially methylated regions were calculated for CpG sites with methylKit installed in R (v3.5) [3]. Differentially methylated cytosines were defined as being present in both biological replicates, having a minimum absolute difference of 1% using the coverage weighted mean, and having an SLIM adjusted *q*value < 0.01 using the methylKit logistic regression model [53]. The change in mean percent methylation (Δme) for all CpG sites within a defined region was calculated by taking the mean number of methylated versus non-methylated CpG sites from the pooled control and treated samples and using Fisher’s exact test FDR < 0.05.

## Supplementary information

**Additional file 1: Table S1.** qRT-PCR primers for RNAseq validation in human genome. **Table S2.** BS-PCR primers for human genome. **Table S3.** BS-PCR primers for human genome. **Figure S1.** RNA sequencing validation with qRT-PCR. **Figure S2.** Pearson’s correlation coefficient for bisulfite sequencing replicates of HEK293. **Figure S3.** Bisulfite sequencing coverage of CpG sites within amplified DNMT1-dependent regions at *HCN2* and *NEFM*. **Figure S4.** Percentage of hyper and hypo differentially methylated CpGs within DNMT1-dependent loci. **Table S4.** CPG Methylation HCN2. **Table S5.** CPG Methylation NEFM.

## Data Availability

The data used to generate this report are available upon request from the corresponding author Dr. Zhibin Wang (zwang47@jhu.edu).

## Abbreviations

PD: Parkinson’s disease
DNMT1: DNA methyltransferase I
ASM: Allele-specific methylation
*HCN2* (gene): Hyperpolarization Activated Cyclic Nucleotide Gated Potassium And Sodium Channel 2 (protein)
*NEFM* (gene): Neurofilament medium polypeptide (protein)
FDR: False-discovery rate
LFC: Log2 fold change
TPM: Transcripts per million
