# Supplementary material for: The conserved DNMT1-dependent methylation regions in human cells are vulnerable to neurotoxicant rotenone exposure
Source: Epigenetics Chromatin. 2020 Mar 16;13:17. doi: 10.1186/s13072-020-00338-8 (PMC7076959; doi:10.1186/s13072-020-00338-8)
Supplement: Supplementary file 1 — Additional file 1: Table S1. qRT-PCR primers for RNAseq validation in human genome. Table S2. BS-PCR primers for human genome. Table S3. BS-PCR primers for human genome. Figure S1. RNA sequencing validation with qRT-PCR. Figure S2. Pearson’s correlation coefficient for bisulfite sequencing replicates of HEK293. Figure S3. Bisulfite sequencing coverage of CpG sites within amplified DNMT1-dependent regions at HCN2 and NEFM. Figure S4. Percentage of hyper and hypo differentially methylated CpGs within DNMT1-dependent loci. Table S4. CPG Methylation HCN2. Table S5. CPG Methylation NEFM. [file 13072_2020_338_MOESM1_ESM.pdf]

Supplementary:

Table S1. qRT-PCR primers for RNAseq validation in human cells

PPFIA4_For	CTCTGCGGATGTTGTCTCCC
PPFIA4_Rev	ATGCTGCCACTGGTTACACG
DNAAF3-For	GGGCTCAAGTCATTCACCCC
DNAAF3-Rev	GGTTGGGCACATGATAGGC
LRRC8D-For	ATGACATTCAGCCAACTTACCG
LRRC8D-Rev	TACTGGCAAACAGACCACCTG
ADRA2C-For	GCCTCAACGACGAGACCTG
ADRA2C-Rev	CCCAGCCCGTTTTCGGTAG
EML2-For	CTCCGTAGCCGTGCTATACAG
EML2-Rev	CCAAGCATTTGATGTCATCGTTG
HCN2-For	AGAAGGGCATTGACTCCGAG
HCN2-Rev	TAGCGGATCAGGCGTGAGA
NEFM-For	GCTCGTCATTTGCGCGAATAC
NEFM-Rev	TTTCTGTACGCAGCGATTTCTAT
GAPDH-For	ACAACTTTGGTATCGTGGAAGG
GAPDH-Rev	GCCATCACGCCACAGTTTC

Table S2. BS-PCR primers for amplicon sequencing in human cells

HCN2-For	TGAGATTATTTTGGTTAATATGGTGAA
HCN2-Rev	CAAAAAAATAAAATTCTTCTTACCTAC
NEFM-For	GGTATTAAGGAGTTTTTGGAG
NEFM-Rev	CTAACCTAACCATTCCCATCTAAAC

Table S3. Gene Ontology cell component enrichment analysis

GO Term	Cellular Component	Gene Ratio	FDR
GO:0005886	plasma membrane	0.008	0.024
GO:0098793	presynapse	0.023	0.028

Table S4. CpG Methylation *HCN2*

CpG site	q-value	Δme
42	6.8E-18	-7.0
54	5.4E-06	-2.6
58	3.3E-04	-1.8
78	1.7E-04	-1.7
140	8.3E-19	-3.9
142	3.6E-13	-3.3
160	2.5E-25	-6.0
164	7.6E-17	-5.0
180	3.0E-13	-4.9

364	5.0E-05	-1.2
406	2.3E-04	1.0
420	1.2E-12	1.7
444	5.4E-06	1.5
450	2.1E-08	-2.7

Table S5. CpG Methylation *NEFM*

CpG site	q-value	Δme
5	6.0E-10	1.6
11	3.2E-09	-1.2
77	7.8E-78	-1.6
89	7.6E-24	1.1
92	8.6E-75	-2.0
96	2.4E-83	-2.3
134	3.4E-36	-1.4
157	2.7E-34	1.6
169	2.9E-22	-1.0
207	7.7E-37	2.7
388	4.0E-04	-2.6
407	2.7E-03	2.1
417	9.5E-11	5.2

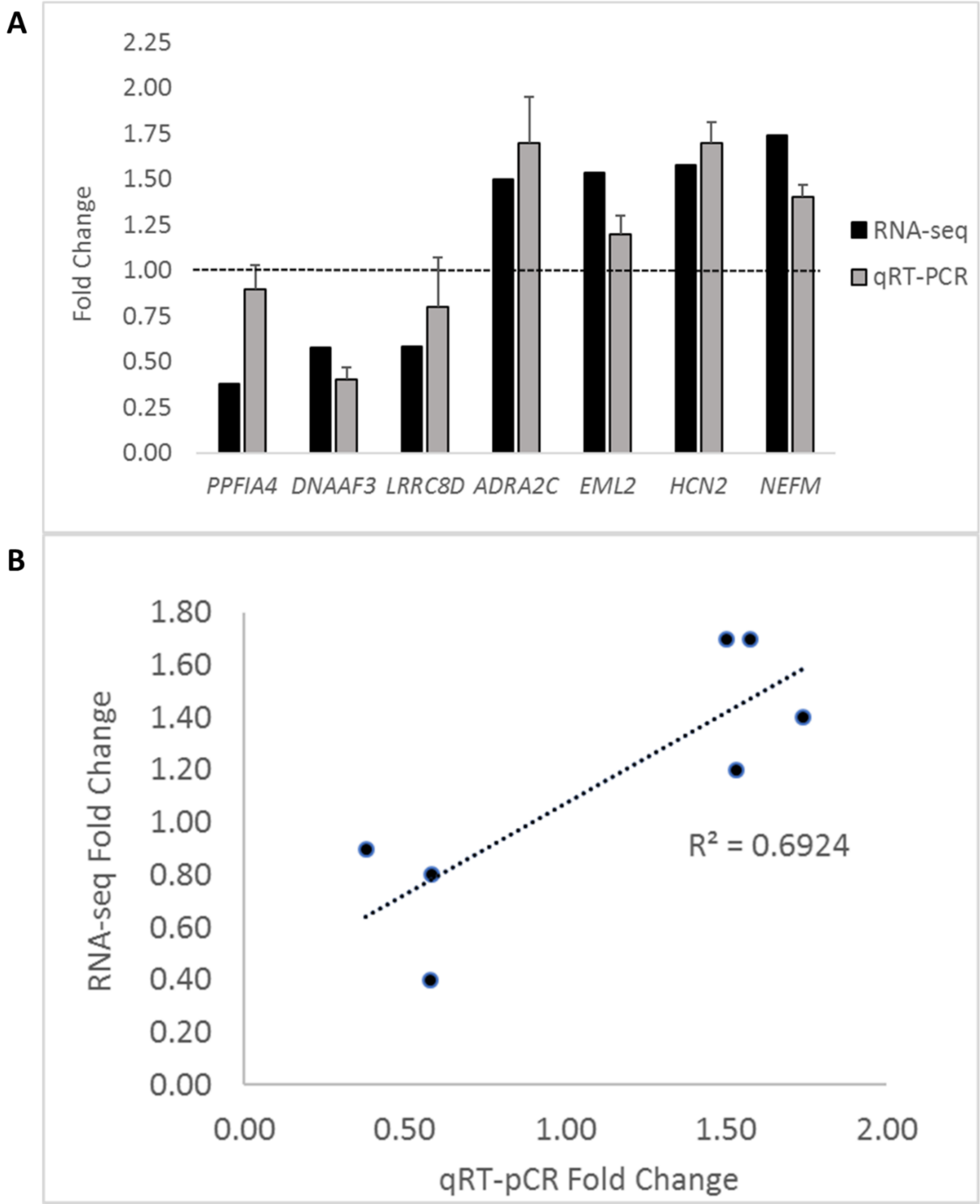

Figure S1. RNA sequencing validation with qRT-PCR. A) Fold change comparison of RNA sequencing results versus qRT-PCR results. B) Linear calibration curve of RNA sequencing results with qRT-PCR results expressed as fold change in expression.

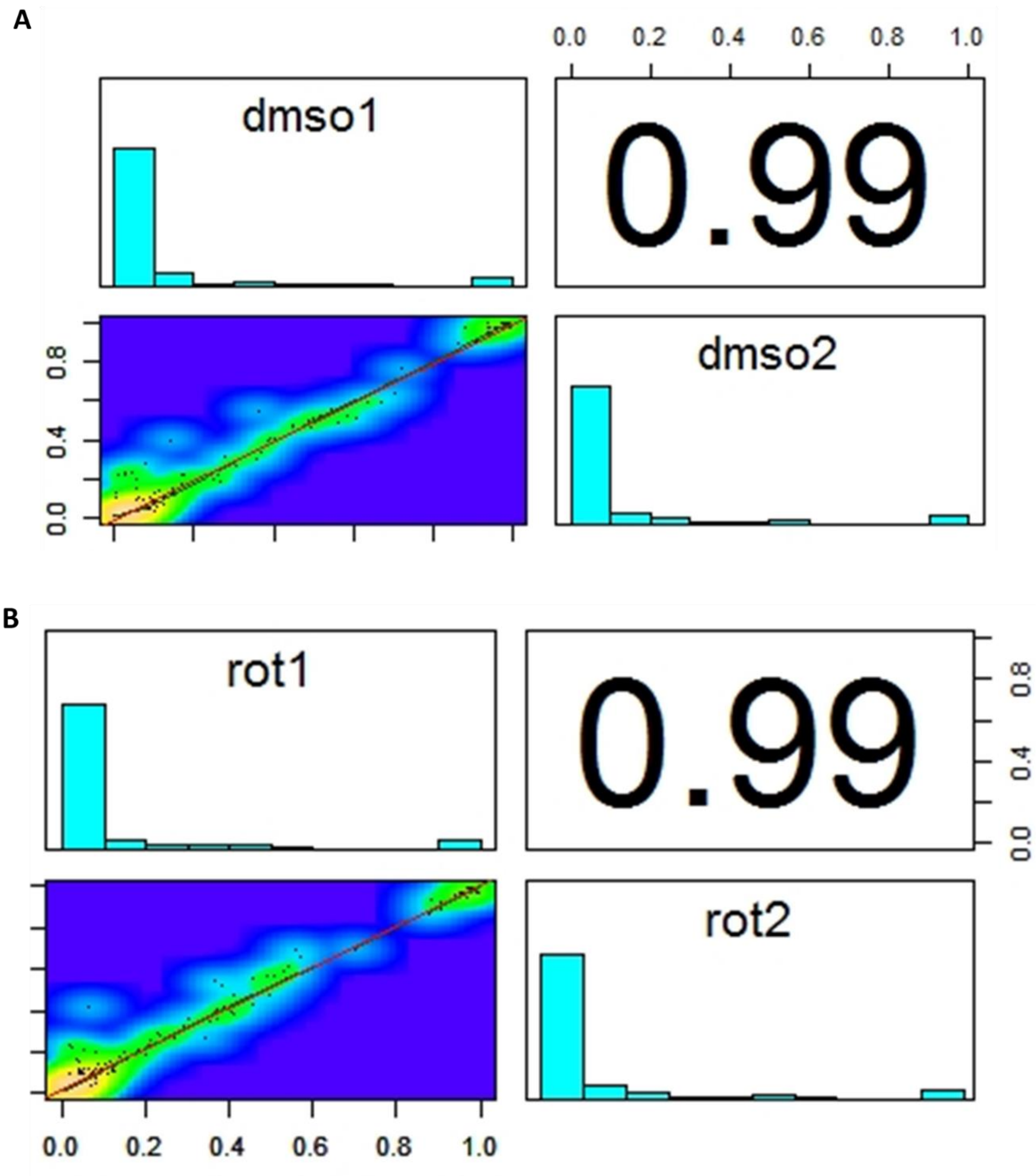

Figure S2. Pearson's correlation coefficient for bisulfite sequencing replicates of HEK293. A) Correlation coefficients of Bisulfite sequencing data between biological replicates of control samples. B) Correlation coefficients of Bisulfite sequencing data between biological replicates of rotenone treated samples.

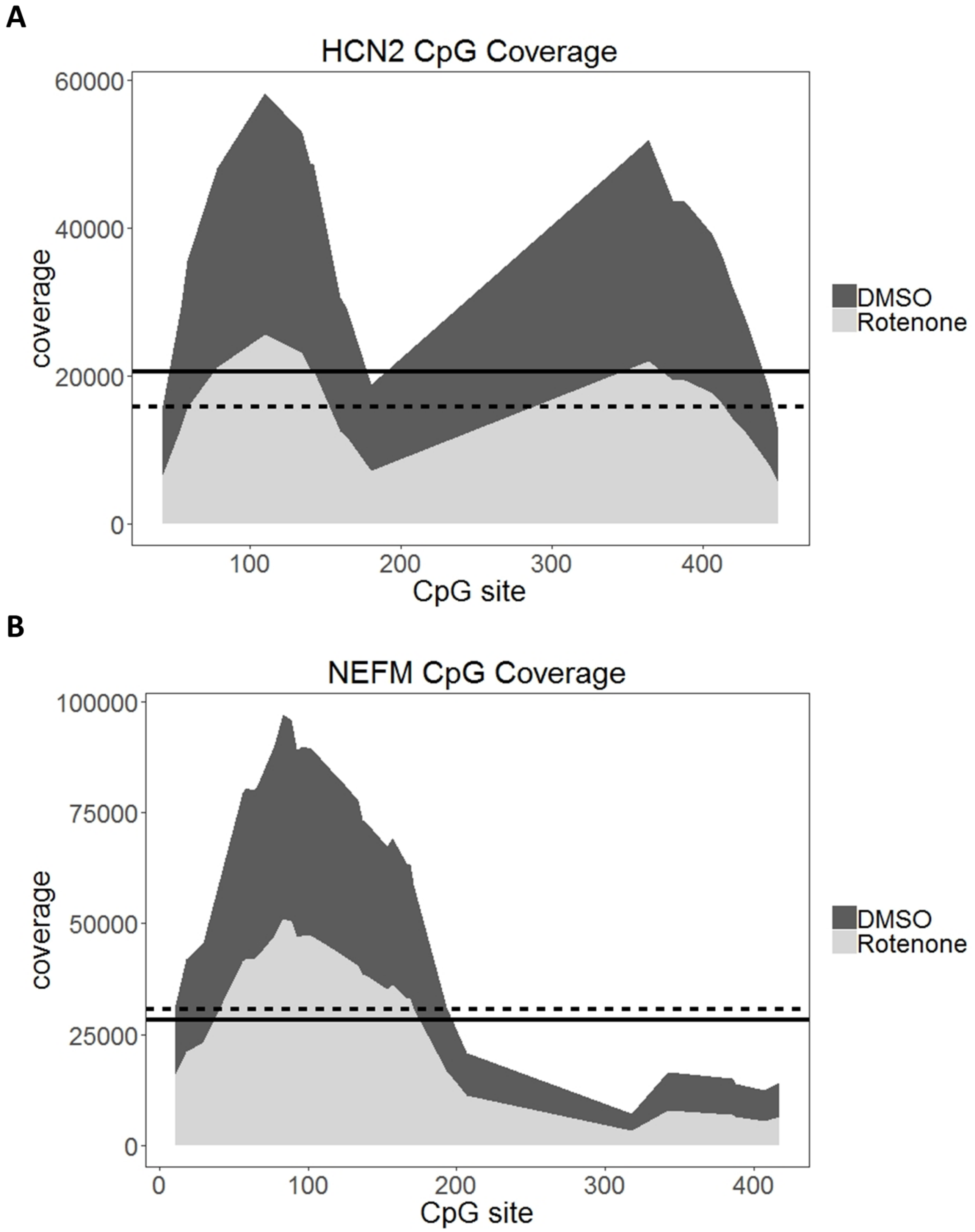

Figure S3. Bisulfite sequencing coverage of CpG sites within amplified DNMT1-dependent regions at *HCN2* and *NEFM*. The average total coverage for all CpG sites within the amplified region is indicated by the straight line for DMSO and the dash line for rotenone.

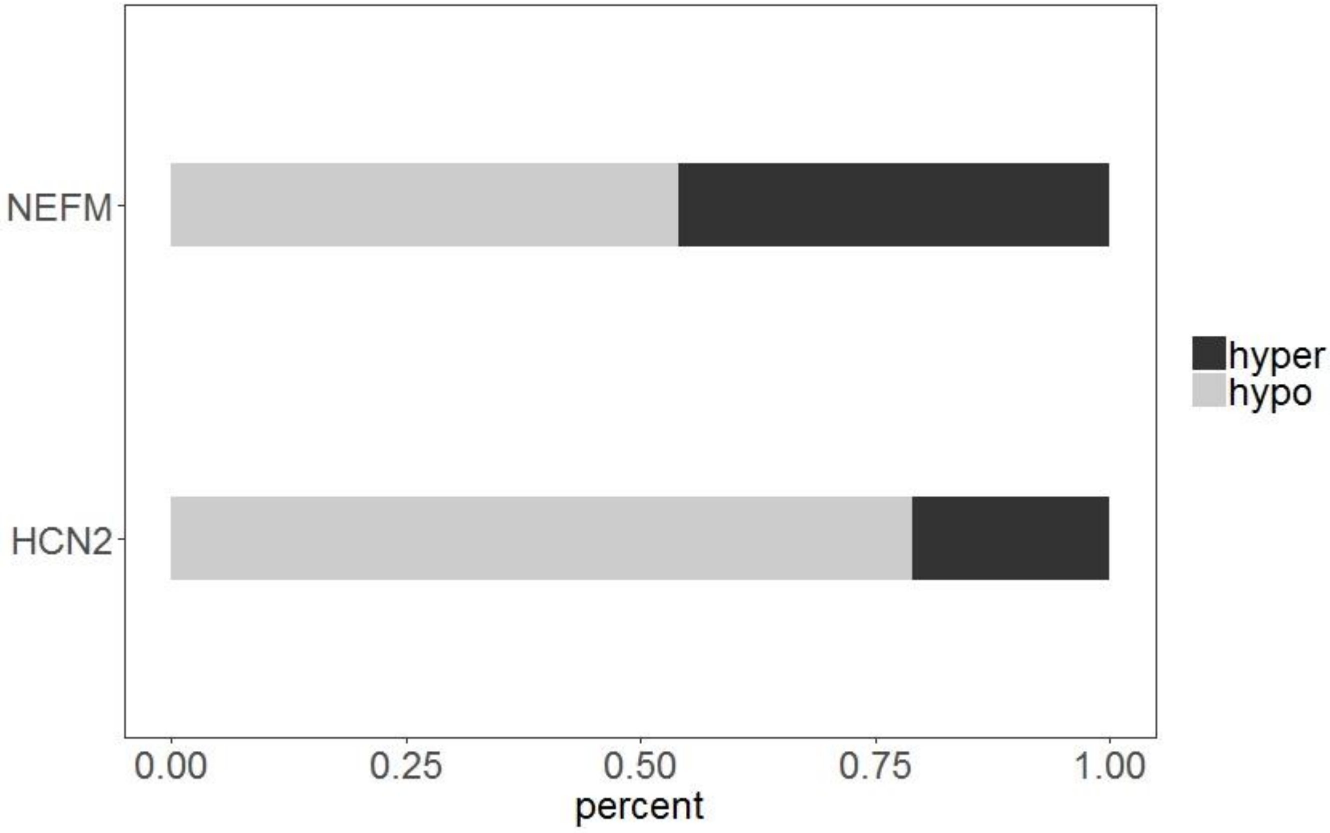

Figure S4. Percentage of hyper and hypo differentially methylated CpGs within DNMT1-dependent loci.
